# Supplementary material for: Extending digital biology: Bacterial survival and morphological heterogeneity under antibiotic stress
Source: iScience. 2026 Oct 31;29(11):118032. doi: 10.1016/j.isci.2026.118032 (PMC13744871; doi:10.1016/j.isci.2026.118032)
Supplement: Supplementary file 1 [file mmc1.pdf]

iScience, Volume *29*

Supplemental information

Extending digital biology: Bacterial survival and morphological heterogeneity under antibiotic stress

Erik Maikranz, Andrey Aristov, Lena Le Quellec, and Charles N. Baroud

Supplementary Figures 1

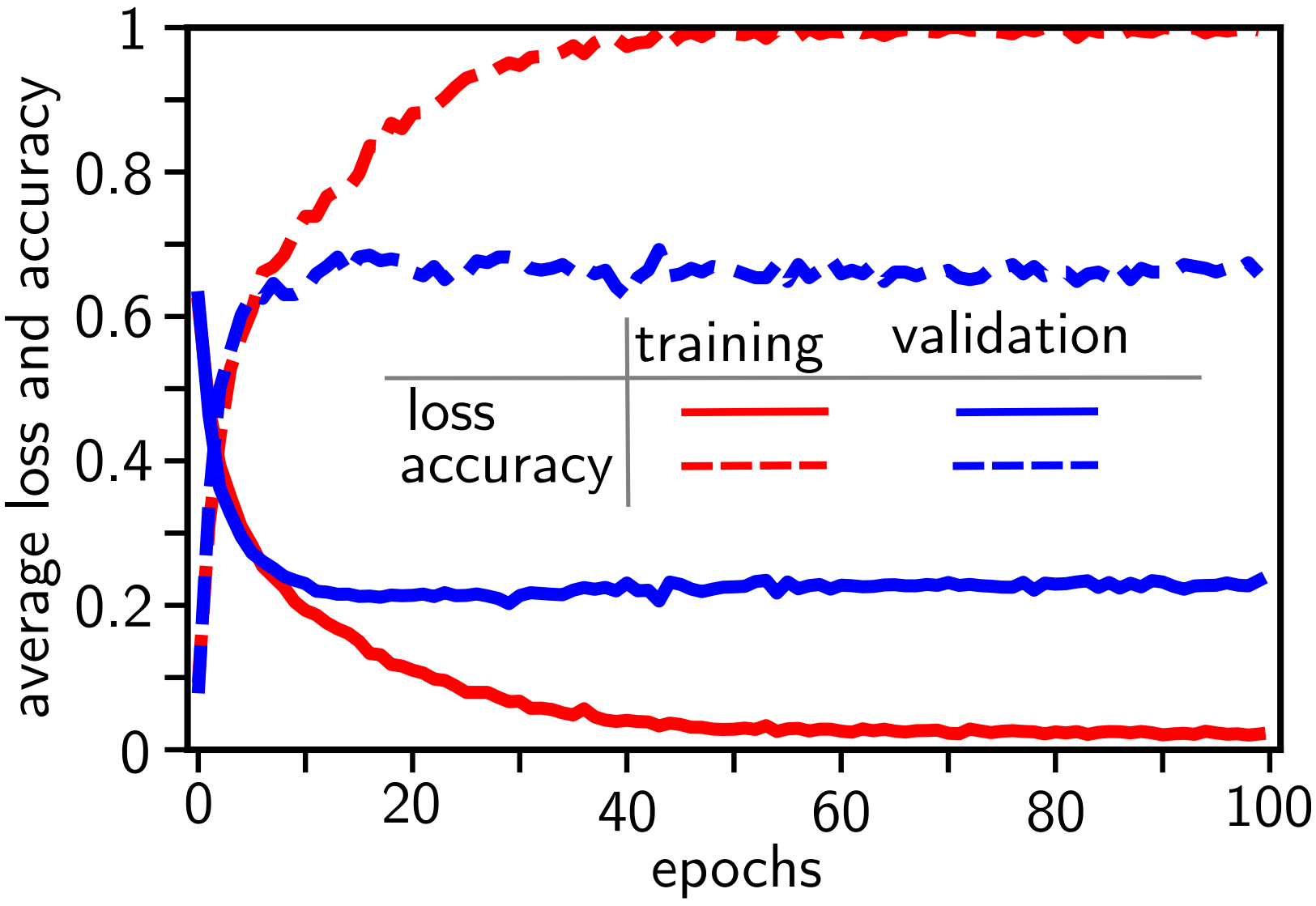

Figure S1: **Supervising the machine learning.** Loss and subset accuracy of test and validation dataset.

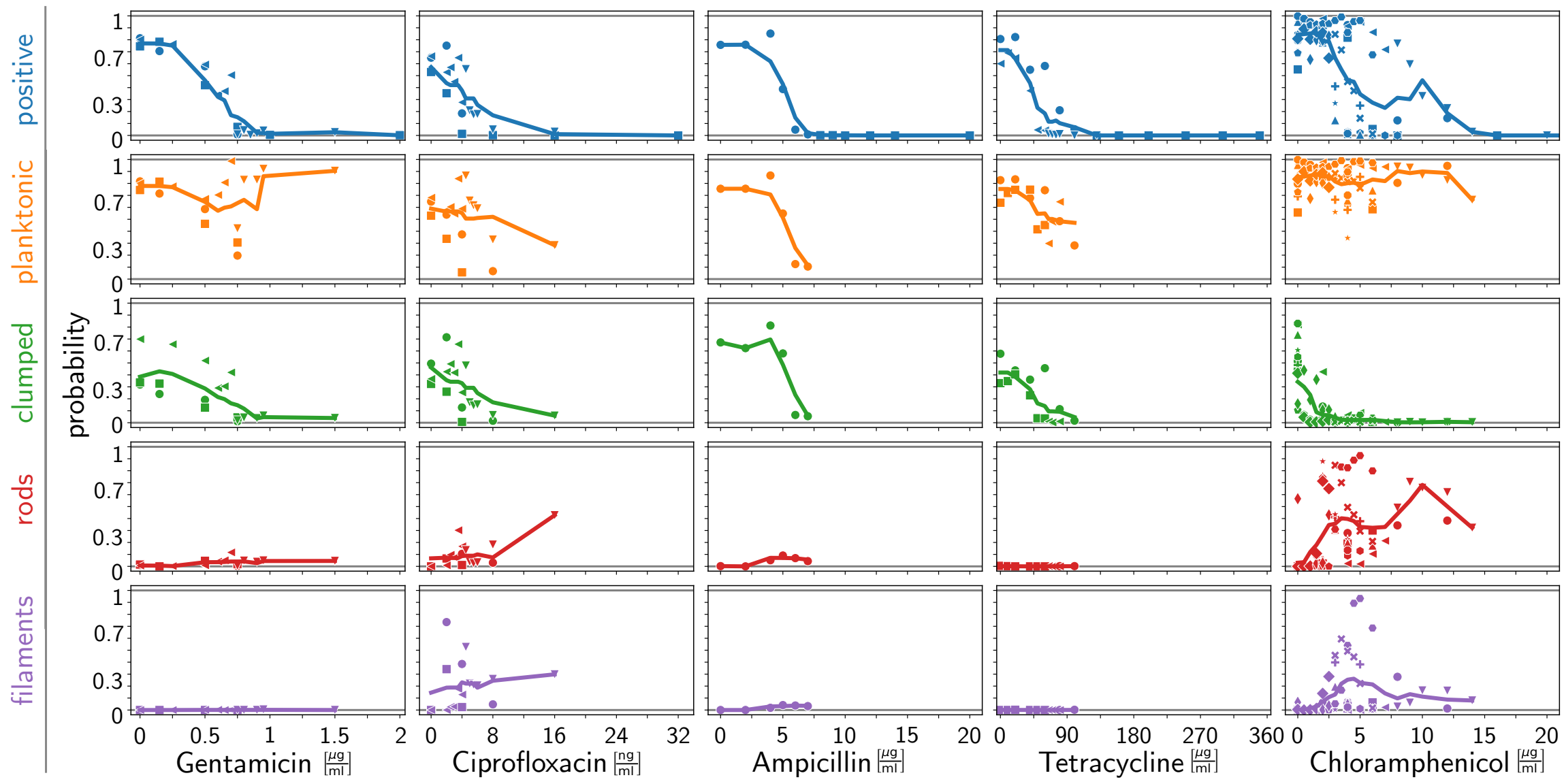

Figure S2: **Unconditioned morphological survival profiles.** Fraction of morphologies as a function of concentration and type of antibiotic. Different symbols correspond to different bacterial cultures, and the solid line shows the moving average with window sizes $(0.3\frac{\mu g}{ml}, 7.8\frac{ng}{ml}, 3.6\frac{\mu g}{ml}, 42\frac{\mu g}{ml}, 2.2\frac{\mu g}{ml})$ respectively. The window sizes corresponds to the weighted concentration averages, with the number of bacterial cultures as weights. Only conditions where the number of positive droplets is larger than two are considered.

Supplementary Tables

antibiotic	MIC
Gentamicin	0.5 μg/ml
Ciprofloxacin	1.25 ng/ml
Ampicillin	4 μg/ml
Tetracycline	64 μg/ml
Chloramphenicol	4 μg/ml

Table S1: **MIC measurements.** MIC as determined from overnight broth microdilution experiments (96 well plates) in LB medium with supplemented IPTG (final concentration 0.05 mM) and a starting concentration of 10^5cells/ml.
